# Limiting Performance of the Ejector Refrigeration Cycle with Pure Working Fluids

**DOI:** 10.3390/e25020223

**Published:** 2023-01-24

**Authors:** Jiawei Fu, Zhenhua Liu, Xingyang Yang, Sumin Jin, Jilei Ye

**Affiliations:** School of Energy Science and Engineering, Nanjing Tech University, Nanjing 211816, China

**Keywords:** ejector refrigeration cycle, working fluids, fluid thermophysical property, thermodynamics

## Abstract

An ejector refrigeration system is a promising heat-driven refrigeration technology for energy consumption. The ideal cycle of an ejector refrigeration cycle (ERC) is a compound cycle with an inverse Carnot cycle driven by a Carnot cycle. The coefficient of performance (*COP*) of this ideal cycle represents the theoretical upper bound of ERC, and it does not contain any information about the properties of working fluids, which is a key cause of the large energy efficiency gap between the actual cycle and the ideal cycle. In this paper, the limiting *COP* and thermodynamics perfection of subcritical ERC is derived to evaluate the ERC efficiency limit under the constraint of pure working fluids. 15 pure fluids are employed to demonstrate the effects of working fluids on limiting *COP* and limiting thermodynamics perfection. The limiting *COP* is expressed as the function of the working fluid thermophysical parameters and the operating temperatures. The thermophysical parameters are the specific entropy increase in the generating process and the slope of the saturated liquid, and the limiting *COP* increases with these two parameters. The result shows R152a, R141b, and R123 have the best performance, and the limiting thermodynamic perfections at the referenced state are 86.8%, 84.90%, and 83.67%, respectively.

## 1. Introduction

**Figure 1:**
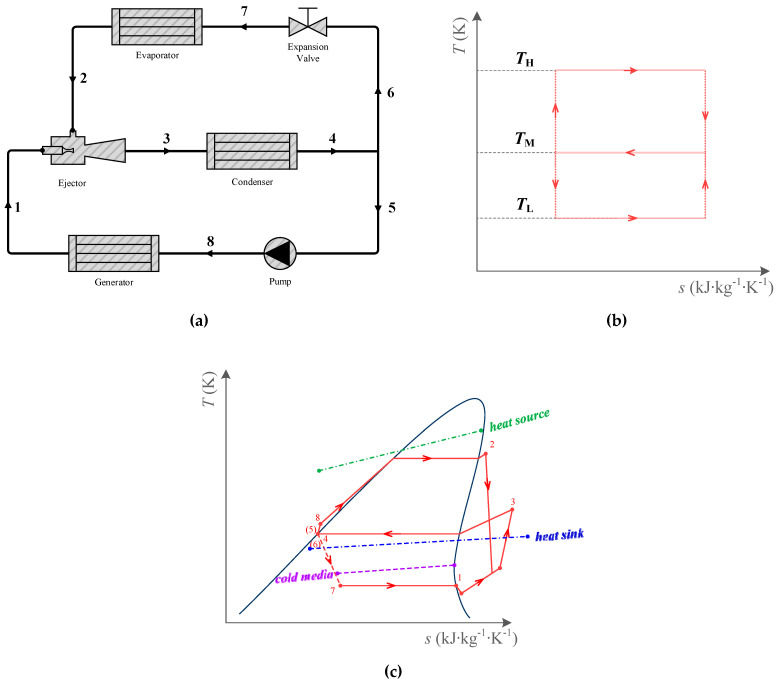
The ejector refrigeration cycle (ERC): (**a**). The schematic for ERC; (**b**). The *T-s* diagram of ERC ideal cycle; (**c**). The *T-s* diagram of practical ERC.

Refrigeration has become a very important part of modern society, and refrigeration consumes more than 20% of the overall electricity used worldwide [1]. Therefore, many scholars have tried to develop non-electric-driven refrigeration systems. Among these cooling technologies, ejector refrigeration cycles (ERCs) are regarded as promising, for their simple structure, lack of moving parts, low capital and maintenance costs, and long lifespan [2]. They can be driven by a low-temperature heat source, but their low-performance coefficient makes it hard to penetrate the commercial market. Compared with the ideal cycle (as shown in Figure 1), the thermodynamics perfection of an actual ERC is less than 50%, according to the statistical results of experimental data [3]. 

An ERC is driven by a heat source. It consists of an ejector, a condenser, a pump, a generator, an expansion valve, and an evaporator, as shown in Figure 1. The ejector is a device that uses high-pressure flow to entrain low-pressure flow for mixing pressure. The working fluid at the outlet of the ejector is condensed in the condenser and divided into two parts. The primary flow is pumped in the pump, evaporates in the generator, and then expands in the ejector nozzle. The secondary flow expands in the expansion valve, and evaporates in the evaporator. Then, the vapor from the evaporator is compressed in the ejector by the primary flow.

For the ideal cycle, all the processes are reversible. Its coefficient of performance (*COP*) is only related to the temperatures of the heat source, heat sink, and cold media. While for an actual ERC, there are different kinds of irreversible losses in it, most of which are related to working fluids. Moreover, its *COP* is not only related to the working conditions, but also to the working fluid properties. Since French engineer Maurice Leblanc introduced the steam ejector refrigeration system in 1910 [4], ERCs have been continuously developing for more than a century. In the past hundred years, a huge number of refrigerants have been applied in ERCs. In the early 1900s, the working fluid was mainly water. Since the 1930s, halocarbon refrigerants have been extensively researched in ERCs both theoretically and experimentally. For example, R11 [5], R12 [6], R113 [7], R123 [5], R134a [8], R141b [9], R142b [10], R152a [11], and R245fa [12]. As the Montreal Protocol on substances that deplete the ozone layer was ratified in 1987, scholars turned their research to natural refrigerants and hydrocarbon refrigerants, such as water [13], ammonia [14], R290 [15], R600 [16], R600a [17], etc. 

To screen the suitable fluids, many scholars have explored the relationship between working fluid thermophysical property parameters and cycle or process performance. Zheng et al. [18] found the parameter ζ of the relative heat loss ratio between a zeotropic mixture and heat transfer fluid. This parameter can reflect the irreversible loss during constant pressure evaporation or condensation, and the parameter can be used as a criterion for the selection of zeotropic working fluids in the heat transfer process. Yang et al. [19] linked the heat transfer process with the thermodynamic behavior of heat exchangers and defined the non-dimensional integration temperature difference of pure working fluid in the heat exchanger. It is useful for the performance evaluation of the heat exchanger. For the compression process of the pump, Xu et al. [20] proposed a parameter *α*_v_*/(ρc_p_)* to identify the influence of the physical properties of working fluid on the performance of the pump. The result showed that the isentropic efficiency of the pump decreases with the increment of *α*_v_*/(ρc_p_)* of different working fluids. For the process of the ejector, Chen et al. [21] found that the slope of the saturated vapor line in the *T-s* diagram of the working fluid has a significant effect on ejector performance. The ejector has better performance when using dry working fluid. Similar results are found in the research from Mwesigye and Dworkin [16]. Buyadgie et al. [22] proposed a criterion of working fluids selection for ERC based on criteria of the significant difference in molecular weights of working fluids. For cycle performance, a fluid with a high molecular weight is a good choice. Kasperski and Gil [23,24] studied the relationship between the normal boiling temperature of refrigerants and system performance. It is found that the refrigerants with lower normal boiling temperatures have better efficiency in the system. Śmierciew et al. [25] proposed a compression efficiency *η*_c_ related to the specific heat of the vaporization of the working fluid and the working pressure used to assess the performance of the ejection refrigeration cycle. It is concluded that the above literature mainly explores the relationship between the practical *COP* of ERC and working fluid properties, under the constraints of different working conditions. To the authors’ best knowledge, scant research has been conducted to investigate the *COP* limit of ERC by considering the working fluid properties.

In this paper, to evaluate the cycle performance upper limit under the constraint of pure working fluids, a limiting ejector refrigeration cycle (LERC) is developed. It is the closest cycle to the ideal cycle that can be achieved by the practical cycle, only taking the influence of working fluid thermophysical properties into account. The *COP* of LERC (*COP*_limit_) represents the maximum *COP* that can be achieved by the actual ERC when only considering the effect of working fluids, and the calculation method of *COP_Limit_* is proposed_._ Moreover, the limiting performance of ERC with different working fluids is analyzed. This paper is organized as follows: in Section 2, the definition of LERC and the *COP*_limit_ methodology are presented. In Section 3, the effects of working fluids and working conditions on the LERC performance are analyzed. In Section 4, the main conclusions are presented.

## 2. Methodology

### 2.1. Fluid Classification and Fluid Slope

In the existing literature, according to the slope of the dry saturated vapor line in the *T-s* diagram, the working fluids are divided into three categories: dry working fluids (dT/ds>0), isentropic working fluids (dT/ds=∞), and wet working fluids (dT/ds<0). Dry or isentropic working fluids are more suitable for the ejector because when wet working fluids expand in the ejector, liquid droplets may be formed, resulting in the performance degradation of the ejector. However, superheat can solve this problem. Strictly speaking, there is almost no isentropic working fluid in pure fluids, but some working fluids are approximately regarded as isentropic working fluids, such as R142b, R1234yf, etc. 

In this research, the slope of the working fluid in a saturated state is obtained by the following method. According to the basic thermodynamic equations, there is

(1) $$ds=\frac{c_{p}}{T}dT-{\left(\frac{\partial v}{\partial T}\right)}_{p}dp$$

When the fluid is in a vapor–liquid two-phase equilibrium state, according to the Clapeyron equation, there is

(2) $$\frac{dp}{dT}=\frac{h_{L-V}}{T_{s}\left(v_{V}-v_{L}\right)}$$

where *h*_L-V_ is specific heat of evaporation, *ν* is the specific volume, and the subscripts V and L refer to saturated vapor and saturated liquid, respectively. Combining Equations (2) and (1), there is

(3) $$ds=\frac{c_{p}}{T}dT_{s}-\frac{\alpha h_{L-V}v}{T_{s}\left(v_{V}-v_{L}\right)}dT$$

where *α* is the thermal expansion coefficient

(4) $$\alpha =\frac{1}{v}{\left(\frac{\partial v}{\partial T}\right)}_{p}$$

Therefore, the slope of the saturated liquid or vapor line in the *T-s* diagram of the working fluid can be expressed as:

(5) $$\beta =\frac{dT}{ds}=\frac{T_{s}}{c_{p}-\frac{\alpha h_{L-V}v}{v_{V}-v_{L}}}$$

**Figure 2:**
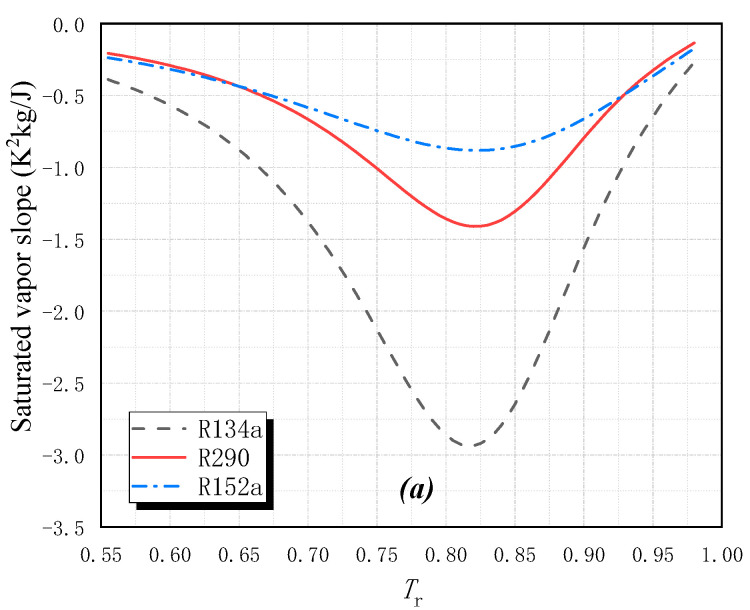

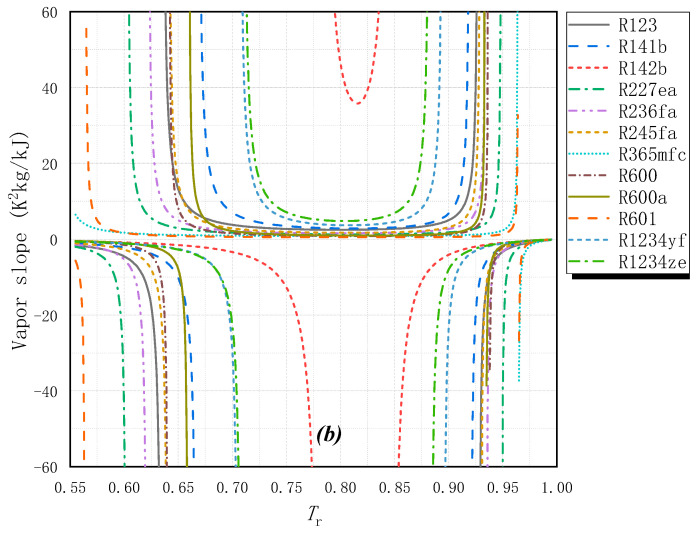
The slope of the saturated vapor in *T-s* diagram for pure fluids: (**a**) wet fluids, (**b**) isentropic fluids and dry fluids.

The saturated vapor slope *β* of 15 pure fluids is shown in Figure 2. For wet fluids, the slope decreases first and then increases as the reduced temperature *T*_r_ (*T*_r_= *T*_s_/ *T*_cr_) increases from 0.555 to 0.995. They all get a minimal value as *T*_r_ is about 0.82. For isentropic fluids and dry fluids, there are two pole points. The slope decreases first and then increases as *T*_r_ increases between these two points, but it decreases as *T*_r_ increases when it is outside this range.

### 2.2. Limiting ERC

To quantify the influence of the fluid thermophysical properties on the cycle performance, the following assumptions are made in this paper:

(1) The heat exchange processes are idealized. The temperature of the heat source and heat sink is constant. In the generator, the highest temperature of the working fluid is equal to the heat source temperature. The condensing temperature is equal to the heat sink temperature, and the evaporating temperature is equal to the cold media temperature.
(2) Irreversibility in other processes of the cycle is ignored, such as the compression process in the pump, and the expansion, mixing, and diffusion process in the ejector are all regarded as isentropic processes, etc.

**Figure 3:**
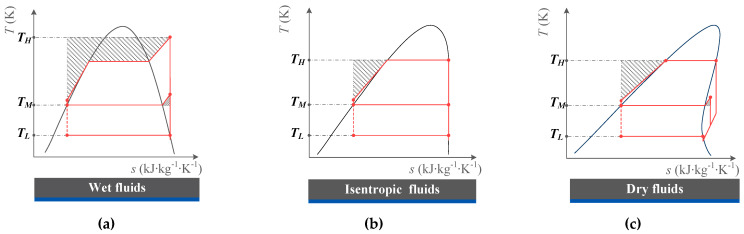
*T-s* Diagram of ERC with different fluids: (**a**) Wet fluids; (**b**) Isentropic fluids; (**c**) Dry fluids.

Under the above assumptions, it can be concluded that the gap between the ideal ERC and actual ERC is caused by the property of the working fluid, as shown in Figure 3, represented by the gray area. It can be seen that the gap is mainly composed of three parts: (1) the heat transfer process in the generator when the fluid is heated from a subcooled state to a saturated liquid state; (2) the heat transfer process in the generator as the fluid is heated from a saturated vapor state to a superheated state; (3) the heat transfer process in the condenser when the fluid is cooled from a superheated gas state to a saturated liquid state. For dry fluid, the loss is caused by (1) and (3). For isentropic fluid, the gap is caused by (1), and for wet fluid, it is caused by (1), (2), and (3).

**Figure 4:**
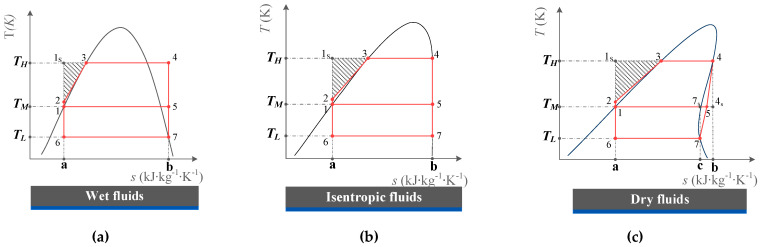
*T-s* Diagram of LERC with different fluids: (**a**) Wet fluids; (**b**) Isentropic fluids; (**c**) Dry fluids.

To evaluate the upper limit of the cycle with actual pure fluid, a cycle that is defined as a limiting ejector refrigeration cycle (LERC) is proposed in this paper. For dry fluids, the irreversible loss in the non-isothermal condensation process is ignored, when it is cooled from superheated gas to saturated vapor. For wet fluids, part of the irreversible loss in the non-isothermal generating process is neglected, when it is heated from saturated vapor to superheated gas. The diagram of LERC for different fluids is shown in Figure 4. Based on the thermodynamic graphical analysis method, the limiting *COP* is obtained.

### 2.3. Limiting COP

For ERC, the *COP* can be expressed as:

(6) $$COP=\frac{{\dot{Q}}_{\mathrm{ev}}}{{\dot{Q}}_{\mathrm{ge}}}=\frac{{\dot{m}}_{\mathrm{ev}}q_{\mathrm{ev}}}{{\dot{m}}_{\mathrm{ge}}q_{\mathrm{ge}}}=\mu \frac{q_{\mathrm{ev}}}{q_{\mathrm{ge}}}$$

where the subscripts ev and ge represent the evaporation process and the generation process, respectively, and *μ* is the entrainment ratio, the flow rate ratio between the secondary fluid and the primary:

(7) $$\mu =\frac{{\dot{m}}_{s}}{{\dot{m}}_{p}}=\frac{{\dot{m}}_{\mathrm{ev}}}{{\dot{m}}_{\mathrm{ge}}}$$

Neglecting the pump power consumption, there is

(8) $${\dot{Q}}_{\mathrm{ev}}+{\dot{Q}}_{\mathrm{ge}}={\dot{Q}}_{\mathrm{co}}$$

(9) $${\dot{m}}_{\mathrm{ev}}q_{\mathrm{ev}}+{\dot{m}}_{\mathrm{ge}}q_{\mathrm{ge}}={\dot{m}}_{\mathrm{co}}q_{\mathrm{co}}$$

According to the mass conservation equation, there is

(10) $${\dot{m}}_{\mathrm{ev}}+{\dot{m}}_{\mathrm{ge}}={\dot{m}}_{\mathrm{co}}$$

Combining Equations (7), (9), and (10), there is

(11) $$\mu =\frac{q_{\mathrm{ge}}-q_{\mathrm{co}}}{q_{\mathrm{co}}-q_{\mathrm{ev}}}$$

When Equation (11) is brought into Equation (6), there is

(12) $$COP=\frac{q_{\mathrm{ge}}-q_{\mathrm{co}}}{q_{\mathrm{co}}-q_{\mathrm{ev}}}\cdot \frac{q_{\mathrm{ev}}}{q_{\mathrm{ge}}}=\frac{q_{\mathrm{ev}}}{q_{\mathrm{co}}-q_{\mathrm{ev}}}\cdot \frac{q_{\mathrm{ge}}-q_{\mathrm{co}}}{q_{\mathrm{ge}}}=\left(\frac{q_{\mathrm{ev}}}{q_{\mathrm{co}}-q_{\mathrm{ev}}}\right)\left(1-\frac{q_{\mathrm{co}}}{q_{\mathrm{ge}}}\right)$$

#### 2.3.1. Wet Fluids and Isentropic Fluids

According to the thermodynamic geometric analysis method, the limiting *COP* can be expressed as an expression of the geometric area as shown in Figure 4. Then, the limiting *COP* can be expressed as

(13) $$CO{P^{\prime }}_{\mathrm{limit}}=\left(1-\frac{{q^{\prime }}_{\mathrm{co}}}{{q^{\prime }}_{\mathrm{ge}}}\right)\left(\frac{{q^{\prime }}_{\mathrm{ev}}}{{q^{\prime }}_{\mathrm{co}}-{q^{\prime }}_{\mathrm{ev}}}\right)=\left(1-\frac{A_{1-5-b-a-1}}{A_{1-3-4-b-a-1}}\right)\cdot \frac{A_{6-7-b-a-6}}{A_{1-5-7-6-1}}$$

where

(14) $${q^{\prime }}_{\mathrm{ev}}=A_{6-7-b-a-6}=T_{L}\left(s_{b}-s_{a}\right)=T_{L}\cdot \Delta s_{a-b}$$

(15) $${q^{\prime }}_{\mathrm{co}}=A_{1-5-b-a-1}=T_{M}\left(s_{b}-s_{a}\right)=T_{M}\cdot \Delta s_{a-b}$$

(16) $${q^{\prime }}_{\mathrm{ge}}=A_{1-3-4-b-a-1}=A_{1_{S}{-4-b-a-1}_{S}}-A_{1_{S}{-3-1-1}_{S}}$$

and $A_{1_{S}-4-b-a-1}{}_{{}_{S}}$ is the input heat for constant temperature heat source:

(17) $$A_{1_{S}-4-b-a-1}{}_{{}_{S}}=T_{H}\cdot \Delta s_{a-b}$$

(18) $$A_{1_{S}{-3-1-1}_{S}}\approx \frac{1}{2\beta }{\left(T_{H}-T_{M}\right)}^{2}$$

where *β* is the slope of the tangent line at state 1 and it can be calculated by formula (5). Although the tangent line does not completely coincide with the saturated liquid line, the difference is very small and neglected. $\Delta s_{a-b}$ is the specific entropy increase in the generating process. Substituting Equations (17)–(21) into (16), there is:

(19) $$CO{P^{\prime }}_{\mathrm{limit}}=\frac{T_{L}}{T_{M}-T_{L}}•\left(1-\frac{T_{M}}{T_{H}-\frac{{\left(T_{H}-T_{M}\right)}^{2}}{2\beta \cdot \Delta s_{a-b}}}\right)$$

#### 2.3.2. Dry Fluids

In the LERC, the condensation heat comes from two parts: one part is carried by the flow from the generator and the other part is carried by the flow from the evaporator. There is

(20) $$Q_{\mathrm{co}}={\dot{m}}_{\mathrm{ev}}q_{\mathrm{co},\;\mathrm{ev}}+{\dot{m}}_{\mathrm{ge}}q_{\mathrm{co},\;\mathrm{ge}}$$

where *q*_co, ev_ is the specific condensation heat from the refrigeration part, and *q*_co, ge_ is the specific condensation heat from the generation part.

Substituting Equations (8), (10), and (20) into (6), the limiting *COP* of wetting fluids can be expressed as

(21) $$CO{P^{\prime \prime }}_{\mathrm{limit}}=\frac{{q^{\prime \prime }}_{\mathrm{ev}}}{q_{\mathrm{co},\;\mathrm{ev}}-{q^{\prime \prime }}_{\mathrm{ev}}}\cdot \frac{{q^{\prime \prime }}_{\mathrm{ge}}-q_{\mathrm{co},\;\mathrm{ge}}}{{q^{\prime \prime }}_{\mathrm{ge}}}=\left(\frac{{q^{\prime \prime }}_{\mathrm{ev}}}{q_{\mathrm{co},\mathrm{ev}}-{q^{\prime \prime }}_{\mathrm{ev}}}\right)\left(1-\frac{q_{\mathrm{co},\;\mathrm{ge}}}{{q^{\prime \prime }}_{\mathrm{ge}}}\right)$$

where

(22) $${q^{\prime \prime }}_{\mathrm{ev}}=A_{6-7-c-a-6}=T_{L}\left(s_{c}-s_{a}\right)=T_{L}\cdot \Delta s_{c-a}$$

(23) $$q_{\mathrm{co},\;\mathrm{ev}}=A_{7s-1-a-c-7s}=T_{M}\left(s_{c}-s_{a}\right)=T_{M}\cdot \Delta s_{c-a}$$

(24) $${q^{\prime \prime }}_{\mathrm{ge}}=A_{1-3-4-b-a-1}=A_{1_{S}{-4-b-a-1}_{S}}-A_{1_{S}{-3-1-1}_{S}}$$

(25) $$q_{\mathrm{co},\;\mathrm{ge}}=A_{4_{s}{-1-a-b-4}_{s}}=T_{M}\left(s_{b}-s_{a}\right)=T_{M}\cdot \Delta s_{b-a}$$

$A_{1_{S}-4-b-a-1}{}_{{}_{S}}$ and $A_{1_{S}-3-1}{}_{{}_{S}}$ can be calculated from (17) and (18). Substituting (22)–(25) and into (21), the limiting COP is expressed as:

(26) $$CO{P^{\prime \prime }}_{\mathrm{limit}}=\frac{T_{L}}{T_{M}-T_{L}}•\left(1-\frac{T_{M}}{T_{H}-\frac{{\left(T_{H}-T_{M}\right)}^{2}}{2\beta \cdot \Delta s_{a-b}}}\right)$$

Comparing (26) and (19), it is found that the expressions of *COP*_limit_ for wet working fluid, isentropic working fluid, and dry working fluid are the same. Therefore, the limiting *COP* of ERC be expressed as:

(27) $$COP_{\mathrm{limit}}=\frac{T_{L}}{T_{M}-T_{L}}•\left(1-\frac{T_{M}}{T_{H}-\frac{{\left(T_{H}-T_{M}\right)}^{2}}{2\beta \cdot \Delta s_{a-b}}}\right)$$

*COP*_limit_ is a function of *T*_H_, *T*_M_, *T*_L_, and *β.* The greater the slope *β* and $\Delta s_{a-b}$, the greater *COP*_limit_.

### 2.4. Limiting Thermodynamic Perfection

For the ideal ERC, the *COP* is

(28) $$COP_{\mathrm{ideal}}=\left(\frac{T_{H}-T_{M}}{T_{H}}\right)\left(\frac{T_{L}}{T_{M}-T_{L}}\right)$$

In this research, a parameter named limiting perfection is proposed, which is defined as the ratio between *COP_ideal_* and *COP_limit_*:

(29) $$\eta_{\mathrm{LTP}}=\frac{COP_{\mathrm{limit}}}{COP_{\mathrm{ideal}}}\times 100\%$$

For actual fluids, *η*_LTP_ can be an index that reflects its distance to “perfection” in the ERC. It can also evaluate the influence of the working fluid itself on the cycle performance upper limit.

## 3. Results and Discussion

Based on the above method, the performance of LERC with 15 refrigerants is researched and compared. These fluids are divided into three groups: wet fluids (R290, R134a, and R152a), isentropic fluids (R141b, R142b, R1234yf, and R1234ze), and dry fluids (R600, R245fa, R600a, R601, R236fa, R365mfc, R123, and R227ea). As mentioned above, there is no perfect isentropic fluid whose slope of saturated vapor in the *T-s* diagram is infinite. Some fluids are approximately regarded as isentropic fluids [26]. In this research, the same method is applied. R141b, R142b, R1234yf, and R1234ze are regarded as isentropic fluids, and the properties of these fluids are listed in Table 1. The effect of operating conditions on *COP*_limit_ and *η*_LTP_ are investigated. The referenced operating conditions of *T*_H_*, T*_L_, and *T*_M_ are 363.15 K, 303.1 K, and 273.15 K, respectively. 

### 3.1. Effect of High Temperature

**Figure 5:**
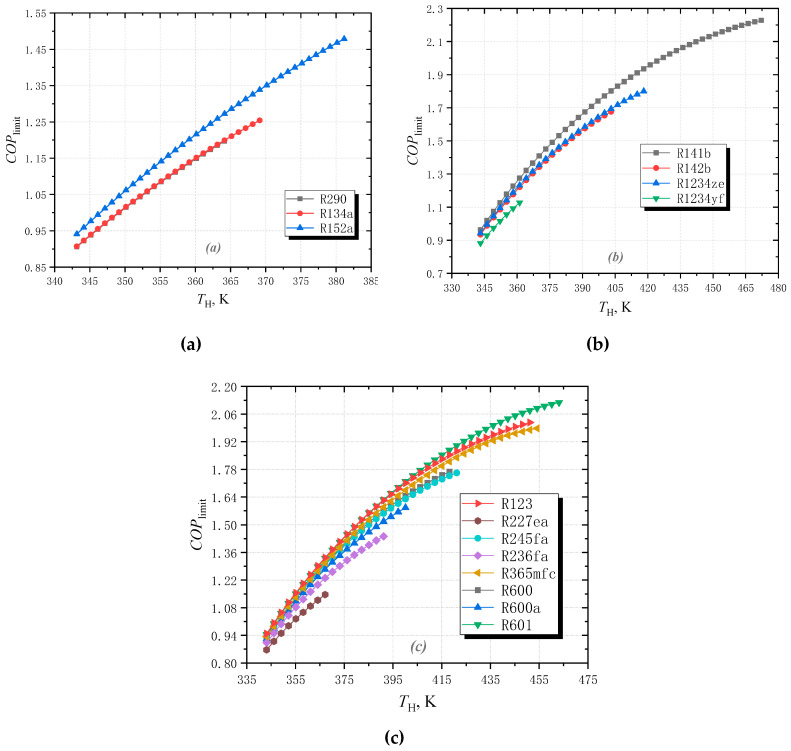
The effect of high temperature on the limiting *COP* with (**a**) wet working fluids; (**b**) isentropic working fluids; (**c**) dry working fluids.

Figure 5 shows the variation in *COP*_limit_ at different high temperatures for these fluids. As can be seen, when *T*_H_ increases from 343.15 to 400.15 K, *COP*_limit_ increases. This is because when *T*_H_ increases, the temperature difference between the middle temperature and high temperature rises. According to Equation (22), the limiting *COP* increases.

As shown in Figure 5a, for wet fluids, *COP*_limit_ of R152a is significantly higher than that of R134a and R290. When *T*_H_ increases from 343.15 to 383.15 K, its *COP*_limit_ increases from 0.941 to 1.479. This is because, for R152a, its Δ*s*_a-b_ is much larger than that of R134a. Although smaller than R290, its slope *β* is greater. For the isentropic fluid group, the *COP*_limit_ of R141b is the largest, and it is the smallest for R1234yf. This is because the slope and entropy increase Δ*s*_a-b_ of R141b is the largest in this group.

For the dry fluid group, as shown in Figure 5c, the *COP*_limit_ of R123 is higher than the others, while it is the lowest for R227ea. When *T*_H_ increases, *COP*_limit_ increases from 0.87 to 1.15 for R227ea. For R123, it increases from 0.95 to 2.02, and for R601, *COP*_limit_ increases from 0.95 to 2.119. The order of *COP*_limit_ for these eight selected dry fluids is R123>R601>R365mfc>R600>R245fa>R600a>R236fa>R227ea. Among these dry fluids, the slope of R123 is the largest, but Δ*s*_a-b_ of R601 is the largest. The slope of R227ea is only smaller than R123, but its Δ*s*_a-b_ is the smallest. The *COP*_limit_ differences between these dry fluids are small at low temperatures, and they increase gradually as *T*_H_ increases. It can be seen from Equation (27) that *COP*_limit_ is a function of high temperature *T*_H_, the slope of the saturated liquid line *β,* and entropy increase Δ*s*_a-b_. For wet fluids and isentropic fluids, *β* and Δ*s*_a-b_ are constant when *T*_H_ varies. The *COP*_limit_ is influenced by *T*_H_. However, for dry fluids, both *T*_H_ and Δ*s*_a-b_ change when *T*_H_ varies. This results in different *COP*_limit_ variations for wet fluids, isentropic fluids, and dry fluids.

**Figure 6:**
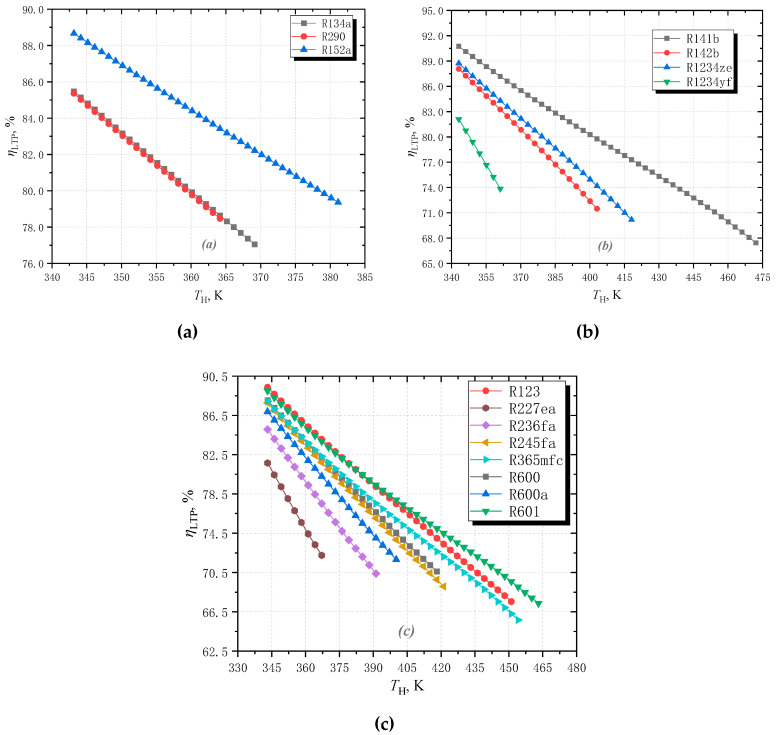
The effect of high temperature on the limiting thermodynamic perfection with (**a**) wet working fluids; (**b**) isentropic working fluids; (**c**) dry working fluids.

Figure 6 shows how the limiting thermodynamic perfection *η*_LTP_ varies with high temperatures for the selected pure fluids. It is found when the *T*_H_ increases, *η*_LTP_ keeps decreasing for all fluids. This is because when *T*_H_ increases, as the slope remains the same, the loss caused by the subcooling section increases. This means that the higher the heat source temperature, the larger the gap between the limiting cycle and the ideal cycle. This also indicates that working fluid has a greater negative effect on cycle performance in higher generating temperatures.

The order of *η*_LTP_ remains the same compared with that of *COP*_limit_ for all fluids. The difference in *η*_LTP_ is caused by that of *COP*_limit_. Therefore, *η*_LTP_ has similar variation law of *COP*_limit_. For the wet fluid group, the order of *η*_LTP_ is R152a>R134a>R290. For the isentropic fluid group, the order is R141b>R142b>R1234ze>R1234yf. For the dry fluid group, the order is R123>R601>R365mfc>R600>R245fa>R600a>R236fa>R227ea. R152a, R141b, and R123 perform best in each group, respectively. The order of *η*_LTP_ is R141b>R123>R152a, with the value of 86.8%, 84.90%, and 83.67%, separately.

### 3.2. Effect of Middle Temperature

**Figure 7:**
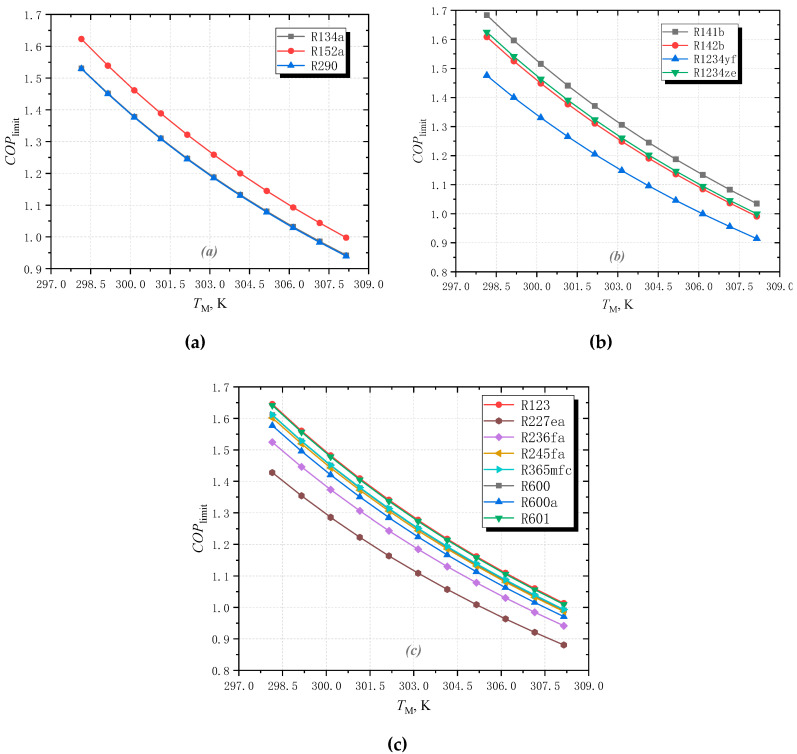
The effect of middle temperature on the limiting *COP* for different fluids: (**a**) wet fluids; (**b**) isentropic fluids; (**c**) dry fluids.

**Figure 8:**
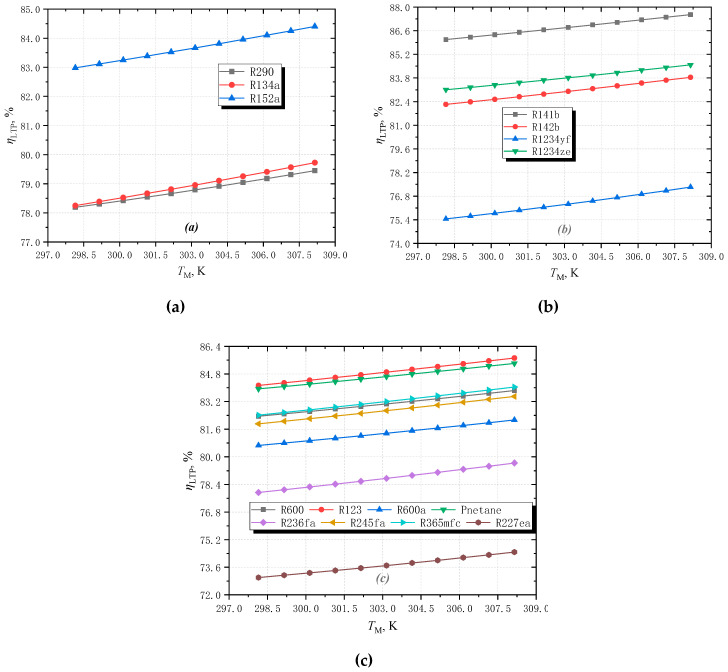
The effect of middle temperature on the limiting perfection with different fluids: (**a**) wet fluids; (**b**) isentropic fluids; (**c**) dry fluids.

Figure 7 and Figure 8 show the effect of middle temperature *T*_M_ on the limiting performance of ERC. It can be seen from Figure 7 that when *T*_H_ increases from 298.15 to 308.15 K, *COP*_limit_ decreases for all fluids. When *T*_M_ increases, the temperature difference between middle temperature and low temperature increases. Consequently, the efficiency of the refrigeration part decreases according to Equation (27). At the same time, the temperature difference between the middle temperature and high temperature is reduced, which leads to a reduction in the efficiency of the cycle driving part. Therefore, the efficiency of the entire refrigeration cycle decreases.

As shown in Figure 8, when the middle temperature increases, the thermodynamic perfection for all fluids decrease. When *T*_M_ increases, the temperature difference between high temperature and middle temperature decreases. As a result, the irreversibility in the subcooling section of the working fluid decreases accordingly.

## 4. Conclusions

To evaluate the performance upper limit of ERC with pure fluids quantitatively, a LERC is proposed in this research. Combined with a thermodynamic graphical analysis method, the limiting *COP* that is expressed by the fluid thermophysical properties and working conditions is derived. And the limiting performance of dry fluids, wet fluids, and isentropic fluids is researched and compared. The key thermophysical parameters of the working fluid that affect *COP*_limit_ are *β* and Δ*s*_a-b_. *COP*_limit_ is a function of *T*_H_, *T*_M_, *T*_L_, *β*, and Δ*s*_a-b_, and *COP*_limit_ increases with the increase in *T*_H_ and *T*_M_ for all fluids; however, *η*_LTP_ decreases as *T*_H_ increases. For the wet fluid group, the *COP*_limit_ and *η*_LTP_ of R152a are the largest. For the dry fluid group, R123 is better than the others, and *η*_LTP_ of R141b, R152a, and R123 at the referenced state is 86.8%, 84.90%, and 83.67%, respectively.

## Figures and Tables

**Table 1:** The properties of the working fluids researched in this work.

Working Fluid		Environmental and Safety Data [27]			Physical Data [28]		Classification	
Refrigerant number	Chemical formula	ODP	GWP 100yr	Safety group	*T*_bo_, K	*T*_cr_, K	Slope *	Type
R134a	CH_2_FCF_3_	0	1370	A1	247.1	374.2	−0.3727	Wet
R152a	CH_3_CHF_2_	0	133	A2	249.1	386.4	−0.4276	Wet
R290	CH_3_CH_2_CH_3_	0	20	A3	231.0	369.9	−0.1225	Wet
R123	CF_3_CHCl_2_	0.01	77	B1	300.9	456.8	2.4578	Dry
R227ea	CF_3_CHFCF_3_	0	3580	A1	256.8	374.9	−1.5428	Dry
R236fa	CF_3_CH_2_CF_3_	0	9820	A1	271.6	398.1	3.6318	Dry
R245fa	CF_3_CH_2_CHF_2_	0	1050	B1	288.2	427.0	1.8363	Dry
R365mfc	CH_3_CH_2_CF_2_CH_3_	0	890	A2	313.3	460.0	0.8124	Dry
R600	CH_3_CH_2_CH_2_CH_3_	0	20	A3	272.6	425.1	0.8897	Dry
R600a	CH(CH_3_)_2_CH_3_	0	20	A3	261.4	407.8	1.3193	Dry
R601	CH_3_CH_2_CH_2_CH_2_CH_3_	0	20	A3	309.2	469.7	0.5027	Dry
R141b	CH_3_CCl_2_F	0.12	717	\	305.2	477.5	3.2231	Isentropic
R142b	CH_3_CClF_2_	0.06	2220	A2	264.0	410.3	−7.1431	Isentropic
R1234ze	CHF=CCF_3_	0	6	A2L	254.1	382.5	−1.0939	Isentropic
R1234yf	CH_2_=CFCF_3_	0	<4.4	A2L	243.6	367.8	−0.2411	Isentropic

* The slope is the saturated vapor slope at 363.15 K.

## Data Availability

Not applicable.

## Nomenclature

Symbols	
*A*	area
*c_p_*	specific heat capacity at constant pressure (kJ∙kg^#x2212;1^∙K^#x2212;1^)
*COP*	coefficient of performance
ERC	ejector refrigeration cycle
*h*_L-V_	specific heat of vaporization (kJ∙kg^#x2212;1^)
LERC	limiting ejector refrigeration cycle
$\dot{m}$	mass flow rate (kg∙s^#x2212;1^)
$\dot{Q}$	heat load (kW)
*s*	specific entropy (kJ∙kg^#x2212;1^∙K^#x2212;1^)
*T*	temperature [K]
*v*	specific volume (m^3^∙kg^#x2212;1^)
*α_v_*	thermal expansion coefficient (1/K)
Greek letters	
*β*	slope of the oblique line
*η*	efficiency (%)
*µ*	entrainment ratio
*ζ*	relative heat loss ratio
*ρ*	density (kg/m^3^)
Subscripts	
bo	boiling
c	compression
co	condensation
cr	critical
ev	evaporation
ge	generation
H	high temperature in cycle
L	low temperature in cycle
limit	performance limit
LTP	limiting thermodynamic perfection
M	Middle temperature
*p*	pressure
r	reduced
s	saturated
V	saturated vapor
L	saturated liquid
